# Expression of *miR-9* and *miR-200c*, ZEB1, ZEB2 and E-cadherin in Non-Small Cell Lung Cancers in Iran

**DOI:** 10.31557/APJCP.2019.20.6.1633

**Published:** 2019

**Authors:** Bahareh Nourmohammadi, Elham Tafsiri, Amirabbas Rahimi, Zahra Noormohammadi, Abolghasem Daneshvar Kakhaki, Wiliam CS Cho, Morteza Karimipoor

**Affiliations:** 1 *Department of Molecular Medicine, Biotechnology Research center, Pasteur Institute of Iran,*; 2 *Department of Biology, Science and Research Branch, Islamic Azad University,*; 3 *Tracheal Diseases Research Center (TDRC), National Research Institute of Tuberculosis and Lung Diseases (NRITLD), Shahid Beheshti University of Medical Sciences, Tehran, Iran,*; 4 *Department of Clinical Oncology, Queen Elizabeth Hospital, Kowloon, Hong Kong, China.*

**Keywords:** microRNAs, miR-9, miR-200c, - non-small cell lung cancer, Iran

## Abstract

MicroRNAs (miRNAs) exert a critical influence on physiological and pathological processes through post-transcriptional modification of their mRNA targets. They play important roles in tumorigenesis and are considered to be potential diagnostic and prognostic biomarkers with various cancers. *MiR-200c* and *miR-9* are regulatory elements that can have dual impacts as oncogenes and/or tumor suppressor genes. MiR-200c regulates two transcription factors, ZEB1 and ZEB2, while *miR-9* is a regulatory factor for the E-cadherin protein which has a critical function in cell-cell junctions and is inhibited by two transcription factors ZEB1 and ZEB2. In this study, expression levels of miR-200c and *miR-9*, ZEB-1, ZEB-2 and E-cadherin were assessed in 30 non-small cell lung cancers (NSCLCs) by real-time qPCR. *MiR-9* was down-regulated significantly in tumor tissues compared to normal adjacent tissues, while there was no significant change in expression level of miR-200c. On the other hand, ZEB1 demonstrated significant increase and ZEB2a decrease at the mRNA level. These results indicate roles for *miR-9* and ZEB1 in genesis of lung cancer, although clinico-pathological associations were not evident. Further studies are necessary to assess implications for treatment of lung cancer.

## Introduction

Lung cancer is the first leading cause of cancer-related deaths worldwide (Ferlay et al., 2015). Itis responsible for 19.4 % of cancer-related deaths every year (Ferlay et al., 2015; Jemal et al., 2011). Over 85% of lung cancer cases are classified as non-small cell lung cancer (NSCLC) (Wu et al., 2012). This includes adenocarcinoma, squamous cell carcinoma, and large cell carcinoma. Adenocarcinoma is the most prevalent subtype and accounts for 40% of all NSCLC patients and squamous cell carcinoma accounts for 25% of NSCLC, whereas large cell carcinoma accounts for 15% of all NSCLC cases. 

Epithelial mesenchymal transition (EMT) is an essential step in tumor cell invasion and metastasis(Tse and Kalluri, 2007). The loss of epithelial markers and gain of mesenchymal morphological features occur when cell adhesion is lost (Fei et al., 2002). Increasing evidence indicated that several regulatory genetic factors involved in the development of EMT. At which miRNAsplay an important role as regulatory elements (Wang et al., 2010). Recent studies in cancer treatment have strong implications for the alterations of miRNAs as one of the most significant markers in human tumorigenesis (Hwang and Mendell, 2006).

miRNAs are short non-coding RNAs that have an important role in many biological processes including tissue differentiation, cell growth and apoptosis (Miska, 2005). The biological miRNA-mRNA interaction can be changed by several factors which are contributed in binding to the target site (Cho, 2011). The most important factor is base pairing between miRNA and target mRNA (or mRNAs). When these elements have perfect complementary to their mRNA targets, mRNA cleavage will occur.According to these studies, miRNAs can have a significant influence on tumorigenesis through post-transcriptional modifications (He et al., 2007). Several studies indicate alteration or loss of miRNAs expression in tumor tissues compared to normal tissues, suggesting that miRNAs can act as either oncogenes or/and tumor suppressors in cancer growth (Gravgaard et al., 2012; Iorio et al., 2005). 

In human cancers, the miR-200 family has been demonstrated to be involved in regulating EMT. This family, which consists of 5 subgroups, including miR-200a, miR-200b, miR-200c, miR-141 and miR-429, has been shown to inhibit EMT (Gregory et al., 2008). Among the targets of miR-200 family, two transcriptional factors ZEB1 and ZEB2 (zinc-finger E-box-binding transcription factors) can have a critical role in promoting EMT by repressing the expression of E-cadherin which is a major adhesion molecule of epithelial cells (Park et al., 2008). In cancer development, the loss of E-cadherin expression is thought to eventuate in increased migratory behavior and metastasis (Dunsmuir et al., 2000; Nass et al., 2000). 

In humans, the mature *miR-9* transcript is generated by three independent genes located on chromosomes 1, 5 and 15. In neural tissues, *miR-9* expression is higher than other tissues (Deo et al., 2006; Ma et al., 2010). Many studies have indicated that *miR-9* has a dual function as an oncogene and tumor suppressor in different type of cancers (Hao-Xiang et al., 2010; Zhu et al., 2012). For example, in breast cancer, *miR-9* promotes EMT by targeting cadherin 1 (CDH1). However, in human melanoma and neuroblastoma *miR-9* has been shown to affect the metastatic mechanism by suppression of multiple genes such as snail1 and MMP-14, therefore overexpression of *miR-9* can inhibit tumor development (Liu et al., 2012; Zhang et al., 2012).

In this study, we measured the expression level of *miR-200c*, *miR-9* and their target genes ZEB1, ZEB2 and E-cadherinin patients with NSCLC.These patients were investigatedby the changes in miRNAsexpression level in tumor and normal tissues and the link between their expression levels with their target genes at mRNA level were analyzed. Indeed, our purpose in this study was the comparison of changes in expression level of miRNAs and their target genesto identify association of these factors with tumorigenesis of NSCLC in Iranian patients.

## Materials and Methods

*Sample collection*

With ethics approval, 30 NSCLC tumor tissues and adjacent normal tissues (2-3 cm farther) of patients undergoing curative surgical resection prior to any kind of treatment in the form of chemotherapy or radiotherapy, including 18 adenocarcinoma and 12 squamous cell carcinoma were collected from 2011 to 2014 at MasihDaneshvari hospital (Tehran, Iran). The clinic pathological features of the patients is summarized at Table 4. The pathological confirmation of all tissue samples were obtained according to standard protocols from pathologists’ of hospital. Written informed consent was taken from all patients before surgical resection and thenfresh tissue samples were transferred immediately to liquid nitrogen and stored at -80^o^C. 

*RNA extraction and cDNA synthesis*

Total RNA was extracted from 15 mg tumor and adjacent normal tissues by using Tripure Isolation Reagent (Roche, Germany) according to the manufacturer’s instruction. The purity and concentration of the extracted RNA was measured by Nanophotometer(260/280 absorbance ratio≥ 1.8). For miRNA analysis, mature miRNA was reverse transcribed using specific stem-loop primers that were designed by our team (Table 1) (Reverted First Strand cDNA Synthesis Kit, Thermo, Lithuania). In brief, 1,000 ng RNA of each sample, 15 pmol stem loop primer, 4 µl 5x buffer, 2 µl DTT, 1 µl dNTP and 1 µl reverse transcriptase enzyme were added in total volume of 20 µl. The thermal cycling condition for the cDNA synthesis was as follows: 37^o^C for 50 min followed by 70^o^C for 15 min. For complementary DNA (cDNA) synthesis of target genes, total RNA was reversely transcribed into cDNA using oligodT primers (RevertAid First Strand cDNA Synthesis Kit, Thermo, Lithuania). In summary, 1,000 ng RNA of each sample, 10 pmololigodT primers, 4µl 5x buffer, 2µl dNTP, and 1 µl reverse transcriptase enzyme were added in the total volume of 20 µl. The thermal cycling condition for the cDNA synthesis was as follows: 42^o^C for 60 min followed by 70^o^C for 5 min. 

*Primer design and qRT- PCR*

Specific forward and universal reverse primers for miRNAs, target genes, and universal probe were designed using GeneRunner and Allele ID 6.0 software in our lab (Table 2 and 3). In this study, for detection of miRNA expression level in tumor and adjacent normal tissues, TaqmanqRT- PCR was performed. All steps were implemented according to the protocols and all samples were performed in triplicate in MicroAmp optical 96-well plate. Then all reactions were normalized with RNU44 control using ABI software (StepOne plus Applied Biosystem USA). In brief, each reaction contains 5 pmol of specific forward and universal reverse primers, 2.5 pmoluniversal probe and 200 ng of cDNA in total volume of 12µl reaction. The thermal cycling condition for the amplification of *miR-200c* and *miR-9* were as follows: initial denaturation at 95°C for 10 min, followed by 45 cycles of 95°C for 15 s and 60°C for 1 min. On the other hand, ZEB1, ZEB2, and E-cadherin expression level was measured by SYBER Premix Ex Taq II (Takara, Japan). mRNA samples were analyzed in duplicates and performed in MicroAmp optical 96-well plate. GAPDH was used as a reference gene using ABI software (StepOne plus, Applied Biosystems). The thermal cycling conditions were as follows: initial denaturation at 95°C for 10 min, followed by 45 cycles of 95°C for 15 s and 60°C for 1 min and 95°C for 15 s. Moreover, the efficiency of amplification for each miRNA and target gene was assessed by serial dilution of cDNA. 

*Data collection and statistical analysis*

The expression level of miRNA and target genes were measured by using the comparative 2^-∆∆CT^ (fold change) method. The statistical significance of relative changes in miRNA and mRNA expression between different groups of lung tumors compared to adjacent normal tissue were determined by ANOVA and T-test (P-value≤0.05). The graphs were created by GraphPad PRISM 5.0 software.

## Results

*Expression level of miR-200c, miR-9 and target genes (ZEB1, ZEB2, and E-cadherin) in tissue samples*

**Figure 1:**
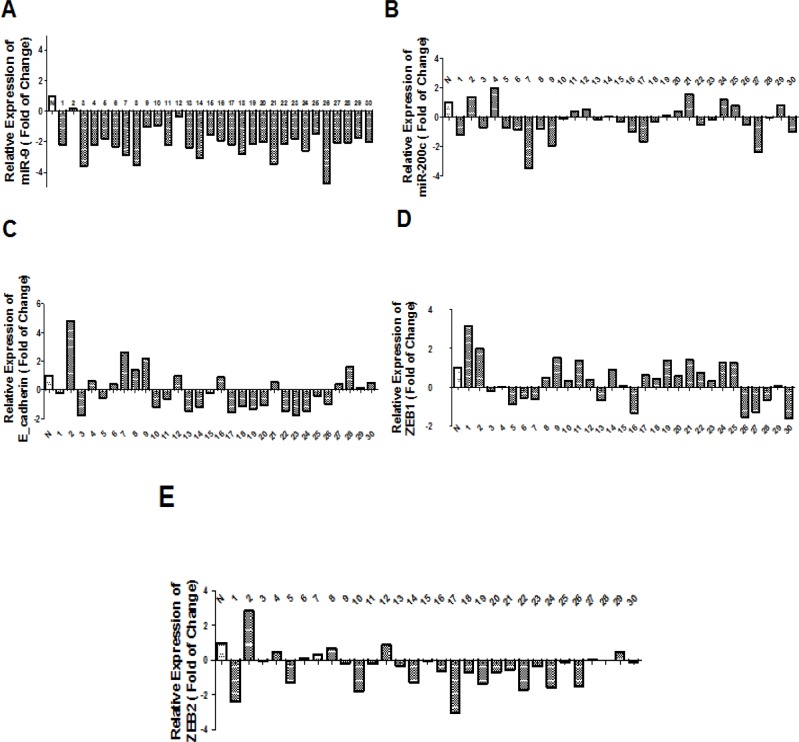
A: miR-9 expression level in NSCLC patients compared to normal adjacent tissue. miR-9 expression was significantly decreased in NSCLC samples compared to normal adjacent tissues (p-value <0.0001). B: Down_regulation of miR-200c was found in patients with NSCLC compared to normal adjacent tissue. (p-value=0.22)C:No significant correlation was found between the expression levels of E-cadherin in patients with NSCLC compared to adjacent normal tissue (p-value=0.82). D:ZEB1 expression level in NSCLC patients compared to normal adjacent tissue. ZEB1 expression level was significantly increased in NSCLC samples compared to normal adjacent tissue (p-value=0.0206). E: Association of ZEB2 expression level in patients with NSCLC compared to adjacent normal tissues. ZEB2 expression level was significantly decreased in NSCLC samples compared to normal adjacent tissues (P value= 0.0207)

In this study, we measured the expression level of *miR-200c*, *miR-9* and target genes (ZEB1, ZEB2 and E-cadherin) as log102^-∆∆CT ^in tumor samples compared to normal adjacent tissues.The target genes were selected by Targetscan and miRwalk databases according to their high score and more complementary base baring between miRNAs seed region and 3’UTR sequence of mRNA of target genes. ZEB1 and ZEB2 were assigned as *miR-200c *target genes and E-cadherin was chosen as *miR-9* target gene at the beginning of the project. According to data obtained in this experiment, downregulation of *miR-9* has been shown in NSCLC patients compared to normal samples (P<0.0001, Figure 1A), although significant downregulation of *miR-200c* in NSCLC samples was less consistent (P= 0.22, Figure 1B).Moreover, we found that the expression level of ZEB1 transcription factor was significantly increased (P= 0.0206, Figure 1D), and the expression level of ZEB2 transcription factor was significantly decreased in patients with NSCLC (P=0.0207, Figure 1E), while the expression level of gene encoding E-cadherin protein did not have a significant change in NSCLC tissues compared to normal tissues (P=0.82, Figure 1C).

*Correlation of miRNAs expression level and subtype*

**Figure 2:**
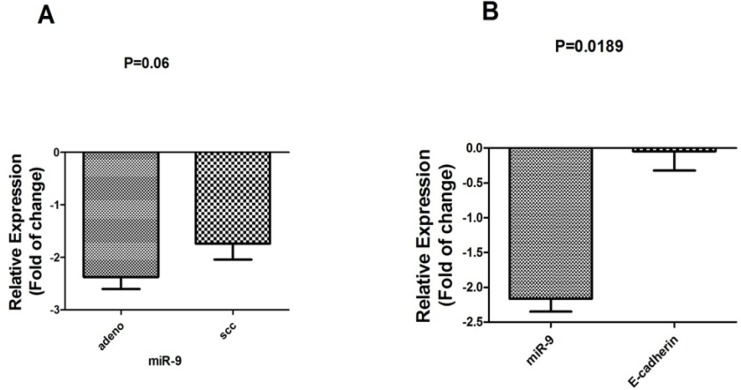
A: miR-9 expression was downregulated in adenocarcinomas (Adeno) subtype compared to squamous cell subtype (SCC) patients. B: Correlation of miR-9 and E-cadherin expression level in all tumor samples (n=30).

Clinical and pathological features of patients with NSCLC and expression level of *miR-200c* and *miR-9* were compared between patients with adenocarcinoma or SCC subtype. Among 30 NSCLC patients, 18 (60%) had a downregulated expression of miR-200c in which 11 out of 18 (61.1%) samples were adenocarcinoma and 7 (38.8 %) were SCC, although there was no significant correlation between the expression level of *miR-200c* and subtype (P=0.55). On the other hand, for *miR-9*, 27 out of 30 (90%) had a downregulated expression of *miR-9*, in which 16 out of 27 (59.2%) samples were adenocarcinoma and 11 out of 27 (40.7%) were SCC (P=0.06, Figure 2A).

*Correlation of miRNAs and genes expression level with clinical and pathological features in NSCLC patients*

In this experiment, we compared the expression level of *miR-9*, *miR-200c* with stage, lymphnode metastasis and smoking. According to data analyzed, no significant correlation was found between the expression level of two miRNAs (*miR-200c* and *miR-9*) and these features. Moreover we measured the expression level of ZEB1, ZEB2 and E-cadherin with stage, histological subtype, lymphnode metastasis and smoking. No significant corralation was found between ZEB1, ZEB2 and E-cadherin expression level and these features (data no shown). 

**Figure 3:**
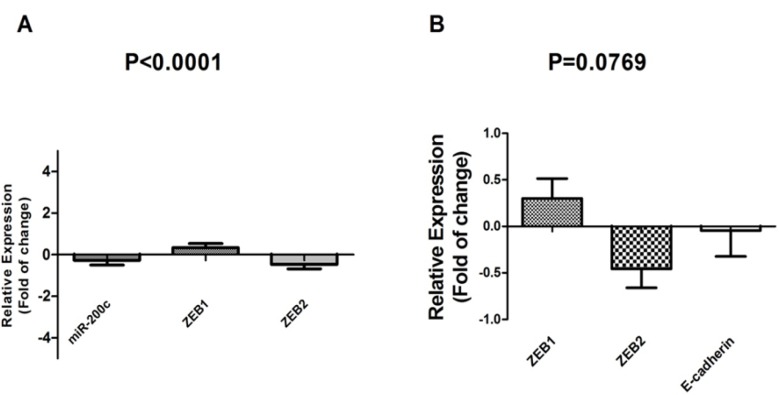
A: Correlation of miR-200c and ZEB family expression level. Down_regulation of miR-200c expression leads to a decrease in ZEB1 expression level. B: Correlation of ZEB family and E-cadherin expression level (ANOVA). Up-regulation of ZEB1is associated with a significant down_regulation E-cadherin expression level (t-test) (p-value<0.05).

**Table 1:** Sequences of Stem-Loop Primers for cDNA Synthesis of miRNAs

miRNA	Length (nucleotide)	%GC	Sequence
miR-9	52	55.8	5´GTCGTATCCAGTGCAGGGTCCGACCGGTATTCGCACTGGATACGACTCATAC3´
Stem-loop			
miR-200c	52	57.7	5´GTCGTATCCAGTGCAGGGTCCGACCGGTATTCGCACTGGATACGACTCCATC3´
Stem-loop			
RNU44	52	57.7	5´GTCGTATCCAGTGCAGGGTCCGACCGGTATTCGCACTGGATACGACAGTCAG3´
Stem-loop			

**Table 2:** Sequences of Primers and Universal Probe for TaqmanqRT-PCR

	Length (nucleotide)	tm	%GC	sequence
miRNA				
miR-9 Forward	21	61	52.4	5´GCG CCG TCT TTG GTT ATC TAG 3´
miR-200c Forward	19	57	52.6	5´ CTG CTT TAA TAC TGC CGG G3´
RNU44 Forward	21	57	42.9	5´ CCT GGA TGA TGA TAG CAA ATG 3´
Reverse	17	55	58.8	5´ TCG TAT CCA GTG CAG GG 3´
Probe	23	66	56.5	5´ -Fam-GACCGGTATTCGCACTGGATACG-Tamra- 3´

**Table 3:** Sequences of Target Gene primers for SYBR Green qRT-PCR

Target genes	Accession number	primer length	Tm	Sequence
ZEB1 Forward	NM-001174096	21	57	5a AGCCAAATGGAAATCAGGATG 3′
ZEB1 Reverse		20	58	5E TTTGGGCGGTGTAGAATCAG 3′
ZEB2 Foeward	NM-014795	20	58	5E AGAAAATGACCTGCCACCTG 3′
ZEB2 Reverse		20	60	5o CTTCATTCTTCTCGTGGCGG 3′
E-cadherin Forward	NM-004360	20	60	5E ACCGAGAGAGTTTCCCTACG 3′
E-cadherin Reverse		21	61	5- CGGAGGATTATCGTTGGTGTC 3′
GAPDH Forward	NM-001289745	21	63	5- TCCACCACCCTGTTGCTGTAG 3′
GAPDH Reverse		21	63	5A ACACCCACTCCTCCACCTTTG 3′

**Table 4:** Clinicopathological Features of NSCLC Patients

Tissue	Case
Age	
< 60	14
≥ 60	16
Sex	
Male	23
Female	7
TNM	
I or II	20
III	10
Subtype	
Adenocarcinoma	18
SCC	12
Pack/year	
-	21
4-120	9
Lymph node metastasis	
Yes	13
No	17

TNM, tumor-node-metastasis

## Discussion

miRNAs are assumed as one of the most important genetic elements which affect carcinogenesisis (Hao et al., 2014; Bryant et al., 2012).These elements exert their influence via post-transcriptional inhibition or degradation of target mRNAs (Souret et al., 2004). Since each miRNA can target multiple genes, it can have an important role in the regulation of tumor development (Akhtar et al., 2016). Recent studies indicate that any changes in the expression of these markers can be associated with EMT and metastatic potential in NSCLC tumors (Jiao et al., 2016; Jin et al., 2016). In this study, the expression level of miR-200c and ZEB family was evaluated and we found that miR-200c was not significantly changed in NSCLC samples compared to adjacent normal tissues. Moreover, we compared the expression level of ZEB family in tumor samples and normal tissues. Our results indicated significant up-regulation of ZEB1 in NSCLC samples and ZEB2 expression level was decreased significantly in NSCLC samples compared to adjacent normal tissues. These results may show a significant role of ZEB1 in the development of NSCLC. According to previous studies, miR-200c has been identified as one of the significant inhibitors of EMT process in breast, bladder and lung cancers (Hilmarsdottir et al., 2014; Liu et al., 2014). Moreover, recent studies have been shown that miR-200c up-regulation has a critical role in the development of various cancer types such as esophageal, colon, ovarian, endometrial and bile duct cancers (Chen et al., 2014; Yeh et al., 2014). These results imply that miR-200c can have a dual function in various type of cancers. For example, Ceppi et al., (2010) demonstrated that low expression of miR-200c was significantly correlated with more lymph node involvement in NSCLC. In addition Zhou et al., (2017) concluded that in gefitinib-resistant cell line, miR-200c was down-regulated compared to its parental cell and transfection of gefitinib resistant cells with miR-200c could lead to significant apoptosis induction by targeting ZEB1 and Pl3k/Akt signaling pathway. Liu et al., (2012) evaluated survival analysis of miR-200c expression in NSCLC samples and they showed that miR-200c was up-regulated in patients with NSCLC compared to normal samples. Moreover, according to several studies, overexpression of miR-200c can lead to ZEB family expression level inhibition and an increase in E-cadherin at mRNA expression level, therefore it can regulate EMT negatively and inhibit invasion capacity of malignant tumors (Korpal et al., 2008; Burk et al., 2008). According to some studies, ZEB1 and ZEB2 transcriptional factors are the activators of EMT process. Indeed, the balance between the expression level of miR-200 family and ZEB family control EMT process (Gregory et al., 2011). Moreover, Hasegawa et al. concluded that among master EMT-inducing factors, ZEB1 transcriptional factor is an important element which is associated with the mesenchymal phenotype in NSCLC cell lines. In addition, they found that ZEB1 knockdown lead to an induction of apoptosis in one of the NSCLC cell lines. These results have been shown that suppressing of ZEB1 expression can be an essential target for therapeutic development in lung cancer (Takeyama et al., 2010). Another microRNA which its expression level is evaluated in this study was *miR-9* and we found that *miR-9* was down-regulated significantly in NSCLC samples compared to adjacent normal samples, so *miR-9* might act as a tumor suppressor in these samples, however, this role needs more functional analysis to confirm tumor suppressive role of *miR-9* in NSCLC. According to some studies Overexpression of this miRNA was reported in brain tumors, colorectal, lung and breast cancers (Nass et al., 2009; Fenger et al., 2014), whereas, down-regulation of this miRNA was identified in gastric, melanoma and ovarian cancers (Khew-Goodall et al., 2010; Tang et al., 2013). These results show that *miR-9* can have a dual function as an oncogene or a tumor suppressor gene in different type of cancers. Based on many studies, E-cadherin which is a protein that mediates cell-cell adhesion might be a target of *miR-9* and loss of this protein could lead to a decrease in cell-cell adhesion and promotes cellular motility, EMT and an increase in the invasion in different type of cancers (Onder et al., 2008). For example, Xu et al., (2017) indicated that the expression level of *miR-9* significantly reduced invasion and metastatic ability in NSCLC cells, while *miR-9* mimic as an inhibitor increased these cells migration activity. *MiR-9* exerted this role via targeting elf5A2, vimentin and E-cadherin. In addition, Bae et al., (2013) concluded that overexpression of E-cadherin has a significant role in the inhibition of EMT process. They demonstrated that by suppression of E-cadherin in the A549 cell line the EMT process could be induced. Moreover, some evidence showed that overexpression of E-cadherin could inhibit converted phenotype and suppresses cell migration in NSCLC (Cui et al., 2015). These results indicate that down-regulation of E-cadherin expression can lead to cancer development. 

In conclusion, *MiR-9* and ZEB2were down-regulated significantly in NSCLC tissues compared to normal adjacent tissues. These results suggesting that *miR-9* might act as tumor suppressor gene in NSCLC samples. While ZEB1expression was significantly increased in NSCLC tissues compared to normal adjacent tissues. ZEB1 might be a therapeutic target in NSCLC patients that needs further experiments in the future (Cho, 2010). Moreover, We found downregulation of MiR-200c in tumor tissues however its expression level was not significant in tumor tissues compared to adjacent normal tissues.
